# Identification of a glutathione transporter in *A. actinomycetemcomitans*

**DOI:** 10.1128/spectrum.03511-23

**Published:** 2023-12-05

**Authors:** Alexander D. Klementiev, Neha Garg, Marvin Whiteley

**Affiliations:** 1 School of Biological Sciences, Georgia Institute of Technology, Atlanta, Georgia, USA; 2 Center for Microbial Dynamics and Infection, Georgia Institute of Technology, Atlanta, Georgia, USA; 3 School of Chemistry and Biochemistry, Georgia Institute of Technology, Atlanta, Georgia, USA; 4 Emory-Children's Cystic Fibrosis Center, Atlanta, Georgia, USA; University of Florida College of Dentistry, Gainesville, Florida, USA

**Keywords:** glutathione, metabolomics, *Streptococcus*, *Aggregatibacter actinomycetemcomitans*, oral biofilm

## Abstract

Bacteria rely on extracellular chemical cues to sense their environment, interact with one another, and shape their chemical landscape. Understanding this biochemistry can help us elucidate the mechanisms by which bacterial interactions impact community function. By applying mass spectrometry to mono- and co-cultures of the oral pathogen *Aggregatibacter actinomycetemcomitans* and the oral commensal *Streptococcus gordonii*, we identified hundreds of extracellular small molecules produced by these bacteria. We discovered that *A. actinomycetemcomitans* secretes millimolar amounts of glutathione and identified a five-gene operon (called *gttABCDE*) that is important for maximum secretion. The metabolomics data set generated in this study provides a valuable data set for unraveling the importance of individual molecules for mediating polymicrobial interactions in the oral cavity.

**IMPORTANCE:**

Microbes produce a large array of extracellular molecules, which serve as signals and cues to promote polymicrobial interactions and alter the function of microbial communities. This has been particularly well studied in the human oral microbiome, where key metabolites have been shown to impact both health and disease. Here, we used an untargeted mass spectrometry approach to comprehensively assess the extracellular metabolome of the pathogen *Aggregatibacter actinomycetemcomitans* and the commensal *Streptococcus gordonii* during mono- and co-culture. We generated and made publicly available a metabolomic data set that includes hundreds of potential metabolites and leveraged this data set to identify an operon important for glutathione secretion in *A. actinomycetemcomitans*.

## OBSERVATION

Streptococci including *Streptococcus gordonii* are found in biofilms throughout the human oral cavity (1). Within these communities, *S. gordonii* produces two extracellular metabolites, L-lactic acid and hydrogen peroxide (H_2_O_2_), at millimolar concentrations. These molecules have been shown to mediate chemical interactions with neighboring oral bacteria. Among the most understood *S. gordonii*-interacting partners is the oral pathogen *Aggregatibacter actinomycetemcomitans*, and these interactions lead to more severe disease than mono-infections (2 – 4). This enhanced pathogenesis has been attributed to the fact that L-lactic acid is the preferred carbon source of *A. actinomycetemcomitans* (5), and its production by *S. gordonii* during co-culture provides *A. actinomycetemcomitans* with a carbon and energy source that allows this relatively slow-growing bacterium to avoid competition with other oral bacteria (2, 5, 6). *S. gordonii*-produced H_2_O_2_ also plays a role in pathogenesis by inducing production of the *A. actinomycetemcomitans* outer membrane protein ApiA (3), which protects *A. actinomycetemcomitans* from complement killing. H_2_O_2_ also plays a role in controlling the biogeography (7) of *A. actinomycetemcomitans-S. gordonii* co-infections by inducing dispersin B in *A. actinomycetemcomitans*, which degrades the biofilm matrix (4).

**Fig 1:**
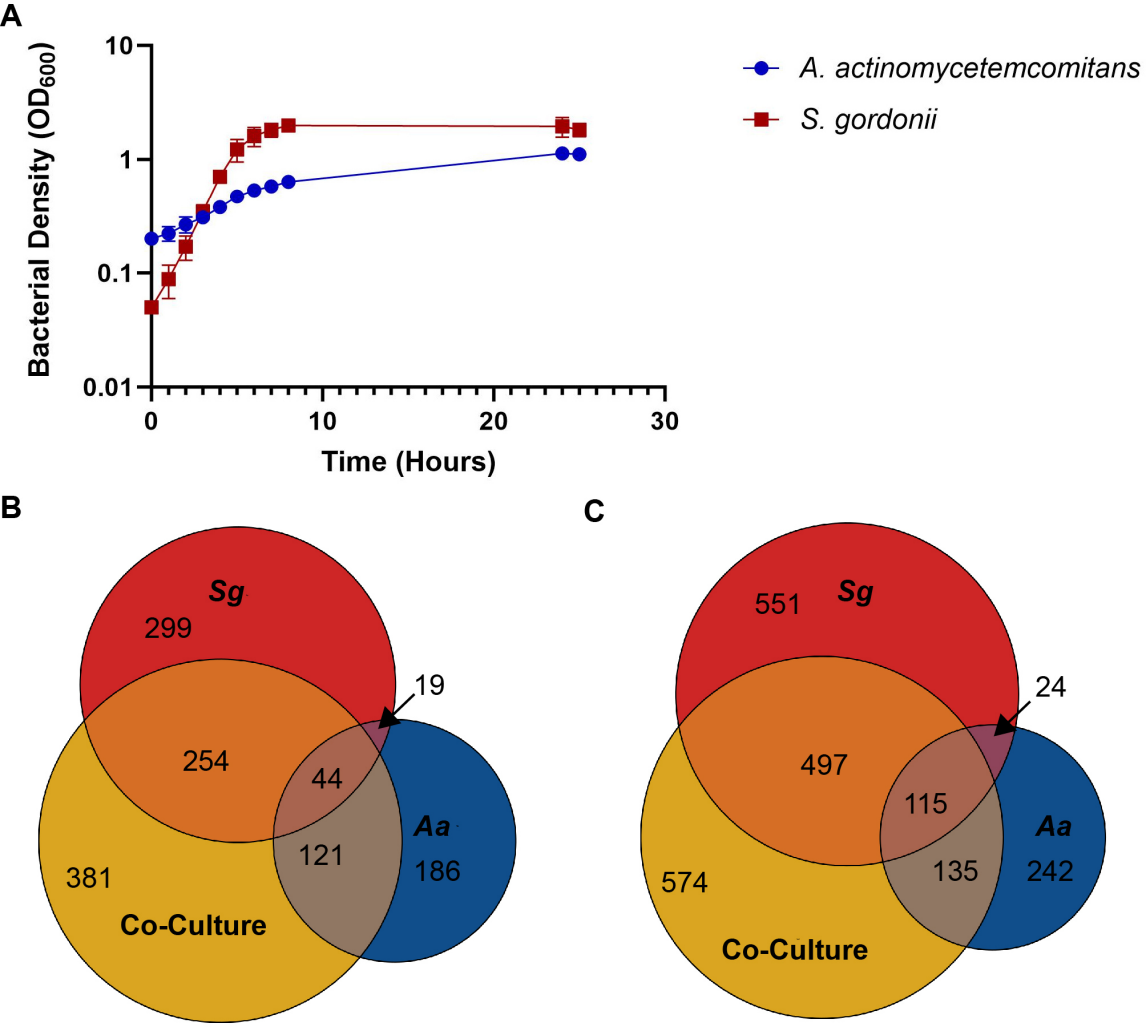
*A. actinomycetemcomitans* and *S. gordonii* produce hundreds of extracellular metabolites. (A) *A. actinomycetemcomitans* and *S. gordonii* mono-culture growth in CDM (*n* = 6). Error bars are standard deviation. (B–C) Mass spectrometry spectral features significantly enriched (*P* < 0.05) in mono- and co-culture growth at (**B**) 3 and (**C**) 5 hours.

While L-lactate and H_2_O_2_ are important metabolic cues mediating interactions between *A. actinomycetemcomitans* and *S. gordonii*, recent genomic work indicates that additional chemical interactions likely occur (8, 9). Here, we used a discovery-based liquid chromatography-mass spectrometry (LC-MS) approach to identify new extracellular chemical cues produced by *A. actinomycetemcomitans* and *S. gordonii*. For these experiments, *A. actinomycetemcomitans* VT1169 and *S. gordonii* DL1.1 (ATCC 49818) were grown planktonically in mono- and co-culture in a chemically defined medium (CDM) (Fig. 1A). CDM is a MOPS-buffered, defined media containing over 60 components, including amino acids, vitamins, and nucleic acids with glucose as the primary energy source (6, 10). Cell-free supernatants were harvested from exponentially growing mono- and co-cultures at 3 and 5 hours and analyzed using LC-MS.

The metabolomics data arising from these LC-MS experiments are metabolite features, each containing a mass-to-charge ratio (*m/z*) and retention time. In addition, fragmentation (tandem) spectra are acquired using a data-dependent acquisition mode. The spectral features (unique retention time and *m/z* pairs) are grouped based on adducts and isotopes and can be identified by matching fragmentation patterns to known molecules in spectral databases. XCMS (https://xcmsonline.scripps.edu/) is a metabolomics analysis platform that extracts the abundances of detected features in mass spectrometry data sets by calculating area under the peak of each chromatogram, which can then be analyzed using a variety of statistical methods. Metabolite features were initially extracted using Compound Discoverer. We further sorted all detected features by *P*-value and fold-change using XCMS and performed identification via spectral matching to an in-house database and METLIN Metabolite and Chemical Entity Database (11). Selectively filtering all spectral features that showed a significant (*P* < 0.05) increase in abundance compared to uninoculated CDM yielded a total of 1,304 and 2,138 features at 3 and 5 hours, respectively, with 863 features shared between the timepoints (Fig. 1B and C). *S. gordonii* produced more unique features in mono-culture compared to *A. actinomycetemcomitans*, with over twice as many (551 vs 242) at the 5-hour timepoint (Fig. 1C). A significant number of features were produced only in co-culture, with 29.2% (381) and 26.8% (574) of total features found in 3- and 5-hour co-cultures, respectively (Fig. 1B and C). These results indicate that both *S. gordonii* and *A. actinomycetemcomitans* produce hundreds of extracellular metabolites during growth in CDM, many of which are only produced in mono- or co-culture.

**Fig 2:**
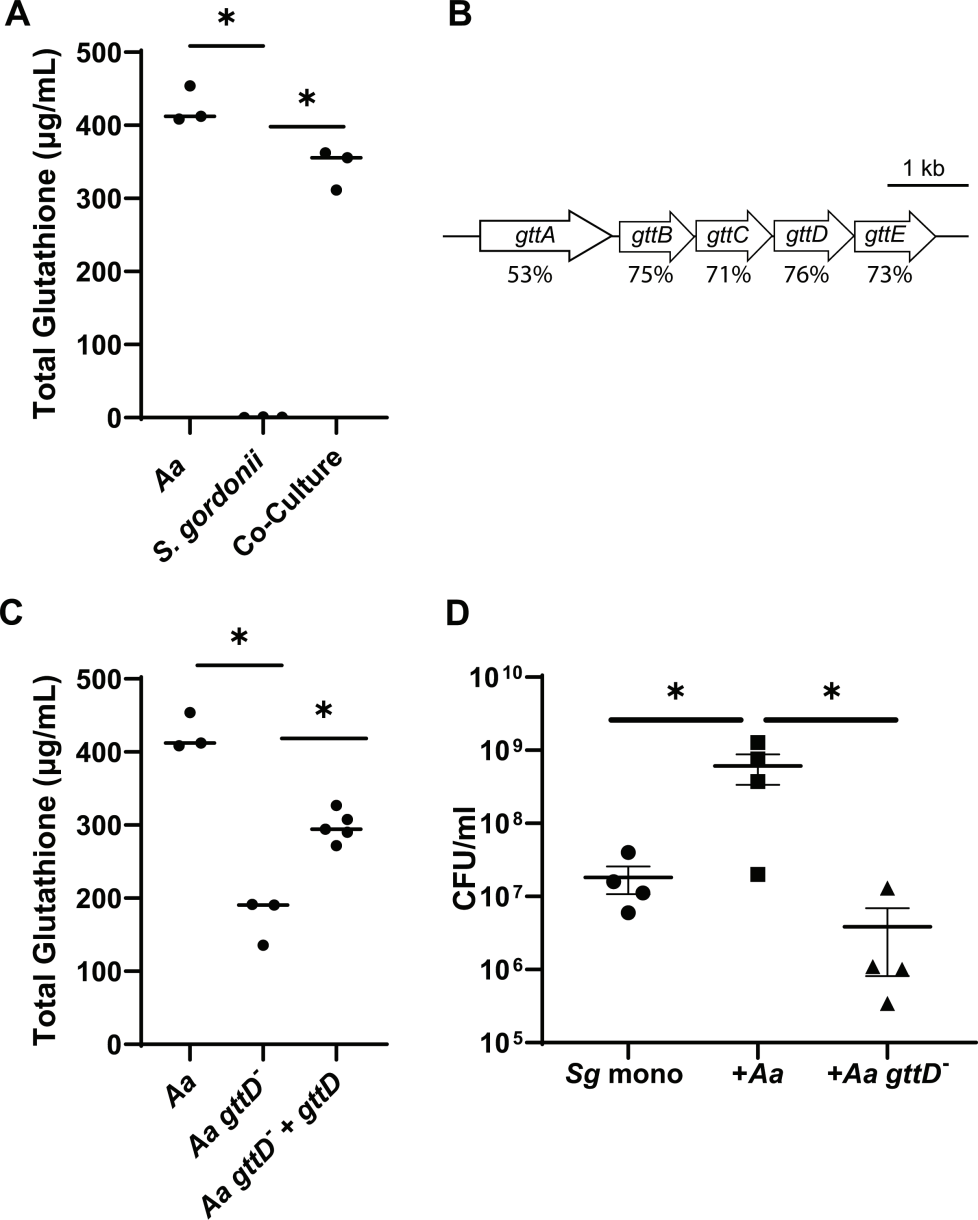
*A. actinomycetemcomitans* secretes GSH, and *gttD* is necessary for maximum secretion. (A) Quantification of extracellular GSH using a fluorometric assay in *A. actinomycetemcomitans* and *S. gordonii* mono- and co-cultures after 5 hours of growth. *S. gordonii* did not produce detectable levels of GSH. (B) Glutathione transporter identified in *A. actinomycetemcomitans*, including percent identity to OppABCDF from *E. coli* MG1655. Operon *gttABCDE* corresponds to loci ACT74_06145 to ACT74_06165 in strain VT1169. (C) Extracellular glutathione produced by wild-type *A. actinomycetemcomitans* (*Aa*)*,* the *gttD* transposon mutant (*Aa gttD^-^*), and the genetically complemented *Aa gttD^−^* (*Aa gttD^−^ + gttD*). (D) *S. gordonii* CFUs during mono- and co-culture with *Aa* or *Aa gttD^−^*. *P < 0.05 using unpaired *t*-test.

From this expansive data set, we selected several molecules for further confirmation by matching retention times to known molecules and/or creating mirror plots of fragmentation spectra using the National Institute of Standards and Technology and Global Natural Products Social Molecular Networking (https://metabolomics-usi.ucsd.edu/dashinterface) databases (Table S1). Based on our interest in glutathione (GSH) (12) and the fact that GSH is a key modulator of virulence in several bacteria (13), we focused on this molecule. GSH is a low-molecular-weight thiol-containing tripeptide (l-γ-glutamyl-l-cysteinyl-glycine) that protects cells from a variety of stresses (13, 14). GSH was detected in *A. actinomycetemcomitans* mono*-* and co-culture supernatants (Table S1). Although the retention time of the analytical standard of GSH matched that from the supernatants, the signal was near the detection limit. Therefore, we confirmed the presence of GSH and compared its abundances across conditions using a fluorometric assay. GSH was present in 5-hour *A*. *actinomycetemcomitans* mono-cultures at 425 µg/mL (1.4 mM) and in 5-hour co-cultures at 343 µg/mL (1.1 mM) but not detectable in *S. gordonii* mono-cultures (Fig. 2A). Thus, high level of extracellular GSH is produced during *A. actinomycetemcomitans* mono-culture and co-culture growth in CDM.

Synthesis of GSH in *A. actinomycetemcomitans* is proposed to occur in a single step via the bifunctional GSH synthase GshF (15). While the biosynthesis of GSH has been studied in *A. actinomycetemcomitans*, transport mechanisms are not known. To identify the mechanism of GSH export, we searched the *A. actinomycetemcomitans* genome for homologs of genes encoding peptide transporters in *Escherichia coli*. This analysis yielded a putative five-gene *A. actinomycetemcomitans* operon containing genes ACT74_06145 to ACT74_06165, which putatively encode proteins with homology to *E. coli* OppABCDF (Fig. 2B). OppABCDF is an ATP-dependent oligopeptide permease that has been well characterized in *Salmonella typhimurium* (16), where it imports peptides containing two to five amino acids. Genes ACT74_06150, ACT74_06155, ACT74_06160, and ACT74_06165 also had homology to the ATP-dependent tetrameric *E. coli* glutathione transporter GsiABCD (17), albeit with lower overall identity (35%–47%). To test whether this operon was important for GSH secretion in *A. actinomycetemcomitans*, a transposon mutant containing an insertion in the *A. actinomycetemcomitans* homolog of *oppD* (ACT74_06160, *gttD*) was obtained from an ordered *A. actinomycetemcomitans* transposon mutant library (18). This mutant grew to similar yields as wild-type (WT) *A. actinomycetemcomitans* but secreted less GSH (Fig. 2C) (18). Expression of *gttD* in *trans* using the plasmid pJAK16 (19, 20) significantly increased the extracellular level of GSH in the *gttD* mutant, although not to the level observed in WT *A. actinomycetemcomitans* (Fig. 2C). This is likely due to either polar effects of the transposon on downstream genes or suboptimal expression of ACT74_06160. These data, along with the fact that transposon mutants in the *A. actinomycetemcomitans oppB* and *oppC* homologs (ACT74_06150 and ACT74_06155, respectively) also reduced extracellular glutathione levels (Fig. S1), designated the ACT74_06145 to ACT74_06165 operon as *gttABCDE* (glutathione transporter A–E).

To assess whether *gttD* is critical for *S. gordonii-A. actinomycetemcomitans* co-culture, we grew these bacteria in mono- and co-culture and then quantified bacterial numbers. Both WT *A. actinomycetemcomitans* and the *gttD* mutant grew to similar levels in mono-culture, and viable cells decreased to below the limit of detection in co-culture (Fig. S2), indicating that *gttD* plays no role in *A. actinomycetemcomitans* co-culture survival. *S. gordonii* numbers increased by over 10-fold during co-culture with WT *A. actinomycetemcomitans* compared to mono-culture, likely due to the ability of *A. actinomycetemcomitans* to detoxify *S. gordonii*-produced H_2_O_2_ (3, 4, 8). However, this increase in *S. gordonii* numbers was not observed during co-culture with the *A. actinomycetemcomitans gttD* mutant (Fig. 2D), indicating that *gttD* is critical for *S. gordonii* survival during *in vitro* co-culture. Since *S. gordonii* can protect host cells from pathogenic bacteria (21), we propose that extracellular glutathione mayalso be beneficial to the host. Ultimately, this study provides a robust metabolomics data set for studying polymicrobial interactions in the future.

## Data Availability

Mass spectrometry raw data are available in the MassIVE public repository DOI: https://doi.org/doi:10.25345/C5PN8XR67.

## SUPPLEMENTAL MATERIAL

Supplemental material spectrum.03511-23-s0001.docx Figures S1 and S2 and additional experimental methods.

Table S1 spectrum.03511-23-s0002.csv Identification of metabolites.

ASM does not own the copyrights to Supplemental Material that may be linked to, or accessed through, an article. The authors have granted ASM a non-exclusive, world-wide license to publish the Supplemental Material files. Please contact the corresponding author directly for reuse.
