# Supplementary material for: Identification of a glutathione transporter in A. actinomycetemcomitans
Source: Microbiol Spectr. 2023 Dec 5;12(1):e03511-23. doi: 10.1128/spectrum.03511-23 (PMC10782972; doi:10.1128/spectrum.03511-23)
Supplement: Supplemental material — Figures S1 and S2 and additional experimental methods. [file spectrum.03511-23-s0001.docx]

**Supplemental Materials**

**FIG S1.** **Mutations in *gttB* and *gttC* reduce extracellular GSH levels.** Quantification of extracellular GSH in supernatants of *A. actinomycetemcomitans*, the *A. actinomycetemcomitans gttB* transposon mutant, and the *A. actinomycetemcomitans gttC* transposon mutant after five hours of growth. * *P* < 0.001 using unpaired t-test.


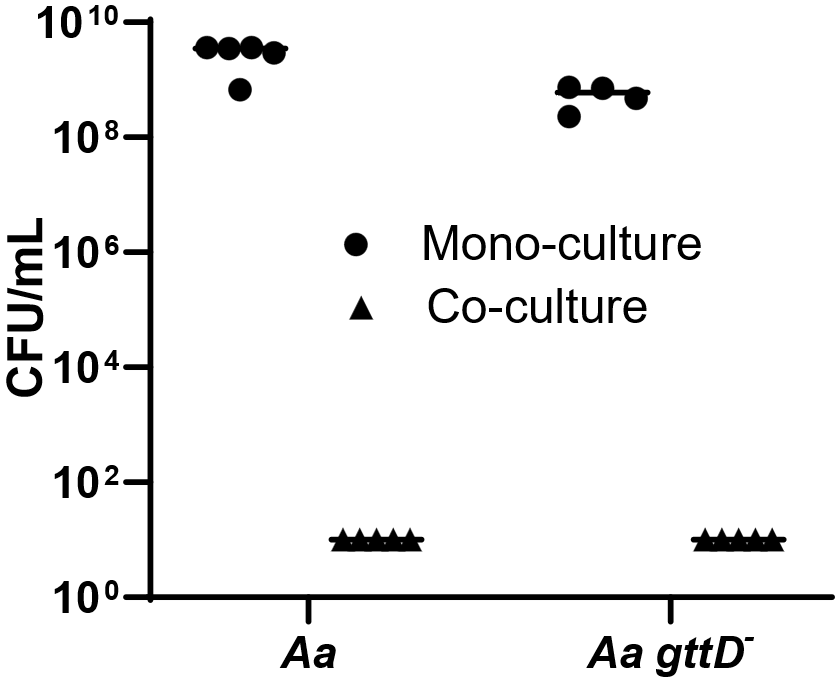


**FIG S2. Mutation of *gttD does not impact A* *actinomycetemcomitans* fitness in co-culture.** *A. actinomycetemcomitans* CFUs during mono- and co-culture with *S. gordonii*. All co-culture CFUs were below the limit of detection (30 CFUs).

**Materials and Methods**

**Strains.** *Aggregatibacter* actinomycetemcomitans VT1169 (1) and *Streptococcus gordonii* DL1.1 (ATCC 49818) were used for all experiments. *Aggregatibacter actinomycetemcomitans* transposon mutants were obtained from an ordered library (2) and confirmed by sequencing.

**Measuring growth of *A. actinomycetemcomitans* in mono- and co-culture with *S. gordonii*.** Bacteria were grown on TSAYE plates overnight, and the following day colonies were grown overnight in CDM. *A. actinomycetemcomitans* cells were back diluted in the morning to an OD_600_ = 0.2 and *S*. *gordonii* cells diluted to OD_600_ = 0.05 and were grown to mid-log phase (OD_600_ = 0.4-0.6). The bacterial cultures were spun down and washed once in CDM. Cell densities were then standardized with OD_600_ = 0.2 for *A. actinomycetemcomitans* and OD_600_ = 0.05 for *S*. *gordonii*. For co-cultures, the final OD_600_ was 0.25 in the final 10 mL volume – 0.2 for *A. actinomycetemcomitans* cells and 0.05 for *S*. *gordonii* cells. Test tubes containing *A. actinomycetemcomitans*, *S*. *gordonii*, or co-culture were allowed to grow at 37°C with 5% CO_2_ and shaking at 4 RPM.

**Mass Spectrometry Sample Preparation.** For mass spectrometry, 700 µL of *A. actinomycetemcomitans*, *S*. *gordonii*, or a co-culture were prepared as above and incubated in a 48-well plate. At 3- and 5-hours, 250 µL of culture was centrifuged for 5 minutes at 16,000 x *g*. 100 µL of supernatant was added to 400 µL 1:3 chloroform/methanol (containing a mix of stable isotope standards: Hypoxanthanine (^13^C_5_, 99%) , L-arginine HCl (^13^C_6_, 99%), hippuric acid (Benzoyl-D5, 98%), and L- methionine (Methyl-^13^C, 99%; Methyl-D3, 98%); 0.7, 13.5, 4.3, and 3.5 µM respectively) in polypropylene tubes. Each sample was vortexed for 5 minutes and centrifuged for 3 minutes at 13,000 x *g* at 4^°^C. 350 µL of the top (methanol/water) layer was transferred to Liquid Chromatography (LC) vials.

**Mass Spectrometry.** LC/MS data were acquired using a Waters Corporation ACQUITY UPLC BEH Amide column (2.1 × 150 mm, 1.7 μm particle size) coupled to a high-resolution accurate mass Orbitrap ID-X Tribrid mass spectrometer. The chromatographic method for sample analysis involved elution with 80:20 water/acetonitrile (MeCN) with 10 mM ammonium formate and 0.1% formic acid (mobile phase A) and MeCN and 0.1% formic acid (mobile phase B) using the following gradient program: 0 min 5% A; 0.5 min 5% A; 8 min 60% A; 9.4 min 60% A; 9.5 min 5% A; and 11 min 5% A. The flow rate was set at 0.4 mL/min. The column temperature was set to 40°C, and the injection volume was 2 μL. The mass spectra were acquired on the Orbitrap ID-X tribrid spectrometer. Full scan and MS/MS data-dependent acquisition (DDA) was performed using the following settings. Full scan data were collected in the positive mode from 70 to 1050 *m*/*z* with a resolution of 240,000. The AGC target was set to 4×10^5^ ions, and maximum ion IT was at 100 ms. DDA for MS^2^ data were collected following the MS scan. MS/MS scans were acquired using an isolation width of 0.8 m/z, precursors were activated with stepped HCD collision energy of 15, 30, 45% or CID 35%, and a dynamic exclusion for 2.5 s.  MS/MS spectra were collected at a resolution of 30000, with an AGC target set at 1×10^4^ions, and maximum ion IT of 100 ms. The raw data files were processed using CompoundDiscoverer 3.0.0.294 (Thermo Fisher Scientific) and manually curated to extract peak areas for the metabolites of interest.

**XCMS Workup and Venn Diagrams.** XCMS (<https://xcmsonline.scripps.edu/>) was used to compare features in all three conditions (*A. actinomycetemcomitans*, *S. gordonii*, or co-culture) vs. media blank. From the output list, we filtered out features with *P* < 0.05 and fold change greater than 2 (increased in each condition). From this list, we aligned features using the R script below by m/z and retention time to create the Venn Diagrams. This analysis was repeated for the three and five-hour time points.

rm(list=ls())

library(ggplot2)

library(tidyr)

library(dplyr)

library(cowplot)

library(gridGraphics)

library(ggpubr)

library(VennDiagram)

library(plyr)

CombinedA <- read.csv(file = 'Bacteria 1 (*A. actinomycetemcomitans*).csv')

CombinedS <- read.csv(file = 'Bacteria 2 (*S. gordonii*).csv')

CombinedCo <- read.csv(file = 'Coculture.csv')

s <- right_join(CombinedCo, CombinedS, by = "mzmed.rtmed", type = "full")

t <- left_join(s, CombinedA, by = "mzmed.rtmed", type = "full")

u <- t[complete.cases(t), ]

nrow(u)

Returns<- subset(t, is.na(t$ID.x))

Returns2 <- subset(Returns, Returns$ID == "AA") #Note ID is the column Title

nrow(Returns2)

##CONFIRM

Check <- subset(t, is.na(t$ID.x))

Check2 <- subset(Check, is.na(Check$ID))

**Verifying Molecules and Mirror Plots.** Molecules were verified using XCMS then confirmed by matching retention times to known molecules and/or creating mirror plots using the NIST database and GNPS (<https://metabolomics-usi.ucsd.edu/dashinterface/>).

**Quantifying GSH.** Cells were prepared as for mass spectrometry. At 5-hours, 1 mL cells were pelleted by centrifugation in a chilled (4^°^C) centrifuge. The supernatant was removed, and total GSH was determined using a GSH Assay Kit (Abcam, ab65322).

**Genetic complementation of the *gttD* transposon mutant.** pJAK16, a broad-host-range plasmid (3, 4), was used for complementation. Plasmid pJAK-*gttD* was constructed by amplifying *gttD* (ACT74_06160) using primers TGACGGTACCGATGCACTCGATCCGAAAGAC (KpnI, underlined) and TGACGAGCTCCAGGTAGCCAACAGTTTCTG (SacI, underlined). The PCR product was gel purified. Both the PCR product and pJAK16 were digested with KpnI and SacI before ligation. The ligation product was transformed into chemically competent *E*. *coli*. Individual colonies were grown overnight, and the plasmid was confirmed by sequencing the entire plasmid. pJAK16-*gttD* was mobilized into *A. actinomycetemcomitans* using conjugation (5) and selected using TSAYE plates containing 3 µg/mL chloramphenicol and 25 µg/mL nalidixic acid.

**References**

1. Mintz KP. 2004. Identification of an extracellular matrix protein adhesin, EmaA, which mediates the adhesion of *Actinobacillus actinomycetemcomitans* to collagen. Microbiol 150:2677-2688.

2. Narayanan AM, Ramsey MM, Stacy A, Whiteley M. 2017. Defining Genetic Fitness Determinants and Creating Genomic Resources for an Oral Pathogen. Appl Environ Microbiol 83:e00797-17.

3. Amarasinghe JJ, Connell TD, Scannapieco FA, Haase EM. 2012. Novel iron-regulated and Fur-regulated small regulatory RNAs in *Aggregatibacter actinomycetemcomitans*. Mol Oral Microbiol 27:327-49.

4. Thomson Valeri J, Bhattacharjee Mrinal K, Fine Daniel H, Derbyshire Keith M, Figurski David H. 1999. Direct Selection of IS903 Transposon Insertions by Use of a Broad-Host-Range Vector: Isolation of Catalase-Deficient Mutants of *Actinobacillus actinomycetemcomitans*. J Bacteriol 181:7298-7307.

5. Ramsey MM, Rumbaugh KP, Whiteley M. 2011. Metabolite Cross-Feeding Enhances Virulence in a Model Polymicrobial Infection. PLOS Pathogens 7:e1002012.
